# Fixed Effects High-Dimensional Profiling Models in Low Information Context

**DOI:** 10.6000/1929-6029.2021.10.11

**Published:** 2021-09-27

**Authors:** Jason P. Estes, Damla Şentürk, Esra Kürüm, Connie M. Rhee, Danh V. Nguyen

**Affiliations:** 1 Mountain View, CA 94043, USA; 2 Department of Biostatistics, University of California, Los Angeles, CA 90095, USA; 3 Department of Statistics, University of California, Riverside, CA 92521, USA; 4 Department of Medicine, University of California Irvine, Orange, CA 92868, USA

**Keywords:** End-stage renal disease, fixed effects, high-dimensional parameters, logistic regression, infrequent events, Firth’s correction

## Abstract

Profiling or evaluation of health care providers, including hospitals or dialysis facilities, involves the application of hierarchical regression models to compare each provider’s performance with respect to a patient outcome, such as unplanned 30-day hospital readmission. This is achieved by comparing a specific provider’s estimate of unplanned readmission rate, adjusted for patient case-mix, to a normative standard, typically defined as an “average” national readmission rate across all providers. Profiling is of national importance in the United States because the Centers for Medicare and Medicaid Services (CMS) policy for payment to providers is dependent on providers’ performance, which is part of a national strategy to improve delivery and quality of patient care. Novel high dimensional fixed effects (FE) models have been proposed for profiling dialysis facilities and are more focused towards inference on the tail of the distribution of provider outcomes, which is well-suited for the objective of identifying sub-standard (“extreme”) performance. However, the extent to which estimation and inference procedures for FE profiling models are effective when the outcome is sparse and/or when there are relatively few patients within a provider, referred to as the “low information” context, have not been examined. This scenario is common in practice when the patient outcome of interest is cause-specific 30-day readmissions, such as 30-day readmission due to infections in patients on dialysis, which is only about ~ 8% compared to the > 30% for all-cause 30-day readmission. Thus, we examine the feasibility and effectiveness of profiling models under the low information context in simulation studies and propose a novel correction method to FE profiling models to better handle sparse outcome data.

## 1. INTRODUCTION

Unplanned readmissions following a hospital discharge are a major source of morbidity and mortality risk for patients on dialysis. The burden of hospitalization is particularly high for patients on dialysis, where the latest U.S. national data shows that the frequency of 30-day readmissions is 31.1%, which is more than double the frequency of readmissions seen in older Medicare beneficiaries without kidney disease (United States Renal Data System/USRDS [1]).

Profiling or evaluation of health care providers, such as hospitals, dialysis facilities, and nursing homes among others, serves multiple purposes, including (1) identifying providers with performance below standard by government agencies for regulatory or payment purposes and (2) conveying information and feedback to stakeholders (e.g., the public, patients, providers) regarding the quality of care among providers. The main focus of our work is objective (1), specifically with respect to the goal of identifying providers whose performances (e.g., 30-day readmission) are exceptionally worse (W) and not different (ND) relative to a reference, such as a national “average” standard. Also related to the inferential process of identifying/flagging providers with 30-day readmission rates W and ND from the national rate, it is of interest to obtain accurate estimates of provider-specific effects and associated quality metrics.

When the outcome, such as 30-day readmission, is not frequent and/or when there are relatively few patients within a provider, referred to as the “low information” context [2], estimation and inference for profiling models are understandably more challenging. This is the situation when the patient outcome of interest is cause-specific 30-day readmissions, such as 30-day readmission due to infections in patients on dialysis, which is only about ~8% compared to greater than 30% for all-cause 30-day readmission. Infection-related hospitalizations are serious adverse events that are oftentimes preventable. Hence, it is an important performance indicator that is carefully monitored in dialysis facilities.

Respecting the data structure that patients are nested within providers, current profiling models for 30-day unplanned hospital readmission are hierarchical logistic regressions of the form *outcome* ~ *provider effects* + *patient case-mix effects*. Thus, patient outcomes vary across providers due to variation in providers’ quality of care (provider-specific effects) and variation in patient-level case-mix effects, which include demographics, comorbidities, and the type of index admission. Because of the nested data structure and the need to stabilize estimation, modeling provider effects as random effects (RE) has been used [2-7].

A justification for the use of RE models is that they provide stable provider effect estimates through shrinkage, although several inherent disadvantages have been noted. In particular, RE estimates are biased toward the overall provider average and biased in the presence of confounding between patient risk factors and provider effects [8]. Also, although the overall *average error* in estimation of provider effects is smaller because mean square error is minimized over the full set of provider effects in the RE approach, fixed effects (FE) estimates have smaller error for outlier ‘providers whose effects are exceptionally large or small’ [8], which are the providers we wish to identify. Our previous works also have shown that the benefit of stabilization comes at a severe cost in substantially biased provider effects estimation and, perhaps more important, at a substantial reduction in the power to identify W providers [9, 10]. Our works and others have used high-dimensional FE models to identify sub-standard (“extreme”) performance, especially for profiling 30-day readmission for dialysis facilities where the outcome is not sparse [3, 8-15]. However, the extent to which FE models are useful in the low information context has not been studied, which is the focus of this work. Thus, we assess the relative performance of the FE model proposed by He *et al*. [15], including the stability of provider-specific estimates and the ability to identify extreme providers in simulation studies. Briefly, the FE model of He *et al*. [15] is a high-dimensional parameter model with a unique fixed intercept for each provider and is used in assessing the performance of dialysis facilities [3, 8, 15]; see also Chen *et al*. [14] and Estes *et al*. [11, 12] for recent dialysis facility profiling applications. Furthermore, in this work, we also propose and examine the performance of a novel corrected FE model estimation approach geared towards estimation under low information context, where the (uncorrected) FE model estimates of some provider-specific effects may be unreliable.

## 2. METHODS: HIGH-DIMENSIONAL FE PROFILING MODELS

We introduce the FE profiling model using the context of hospital readmission as an illustrative example. Let the binary outcome *Y_ij_* equal 1 if patient index discharge *j* in provider *i* results in a readmission within 30 days, for patient discharge *j* = 1,2,…,*N_i_* in provider (dialysis facility) *i* = 1,2,…,*F*. The FE profiling model (He *et al*. [15]) is

(1) $$g(\mu_{ij})=\gamma_{i}+\beta^{T}Z_{ij},\;\;\;i=1,\ldots ,F,$$

where *γ* = (*γ*_1_,…,*γ_F_*) are the provider-specific fixed effects, *μ_ij_* ≡ *E*(*Y_ij_* ∣ *Z_ij_*) = Pr(*Y_ij_* = 1 ∣ *β*,*γ_i_*,*Z_ij_*) = *p_ij_* is the expected readmission for patient index discharge *j* = 1,2,…,*N_i_* in provider *i* = 1,2,…,*F*, and *g*(*p_ij_*) = log{*p_ij_*/(1 − *p_ij_*)} is the logit function. In profiling model (1), the *r* patient risk adjustment factors for discharge *j* in provider *i* are denoted by the covariate vector *Z_ij_* = (*Z_ij_*_1_,…,*Z_ijr_*)*^T^* corresponding to parameters *β^T^* =(*β*_1_,…,*β_r_*). In practice, the process of risk adjustment is complex and depends, in part, on policy objectives and the specific patient population (e.g., general population, dialysis population). However, we point out that it is critical to adequately risk-adjust for patient-level factors and *avoid* inclusion of variables (e.g., provider-level or patient-level variables) that are/may be related to the process of care (e.g., see [2, 3, 13]).

To avoid confusion, we emphasize that the model shown in (1) is not a collection of individual models (i.e., not a separate model for each provider), but rather a single model with high-dimensional parameters and requires simultaneous estimation for thousands of provider-specific effects/parameters ($\{\gamma_{i}{\}}_{i=1}^{F}$ and *β*). For example, for profiling dialysis facilities the dimension of *γ* = (*γ*_1_,…,*γ_F_*)*^T^* is > 6,000 dialysis facilities across the U.S., and the dimension of *β* is ~ 40. Standard estimation (e.g., maximum likelihood) and software fails; thus, He *et al*. (2013) proposed a feasible estimation method based on an alternating one-step Newton-Raphson that iterates between estimation of *β* and *γ_i_*.

The summary performance index for each provider which incorporates patient-level risk factors (*Z*’s) used in practice is the standardized readmission ratio (SRR). For FE model (1), given the provider and the patient case-mix effect estimates, denoted by ${\hat{\gamma }}_{i}$ and $\hat{\beta }$, respectively, the estimated SRR for provider *i* is

(2) $$SRR_{i}=\frac{\sum_{j=1}^{N_{i}}{\hat{p}}_{ij}}{\sum_{j=1}^{N_{i}}{\hat{p}}_{M,ij}},$$

where ${\hat{p}}_{ij}=g^{-1}({\hat{\gamma }}_{i}+{\hat{\beta }}^{T}Z_{ij})$ is the estimated probability of readmission for patient *j* in provider *i* and ${\hat{p}}_{M,ij}=g^{-1}({\hat{\gamma }}_{M}+{\hat{\beta }}^{T}Z_{ij})$. The aggregate parameter ${\hat{\gamma }}_{M}$ in the denominator is taken to be the median of the $\{{\hat{\gamma }}_{i}{\}}_{i=1}^{F}$. Thus, the numerator of *SRR_i_* is the expected total number of readmissions for provider *i* and the denominator is the expected total number of readmissions for an “average” facility (taken over the population of all providers), adjusted for the particular case-mix of the *same* patients in provider *i*. Note that *SRR_i_* estimates the true quantity $\overset{\tilde{}}{SRR}{\;}_{i}=\sum_{j=1}^{N_{i}}p_{ij}\;∕\;\sum_{j=1}^{N_{i}}p_{M,ij},\;\text{where}\;p_{M,ij}=g^{-1}(\gamma_{M}+\beta^{T}Z_{ij})$.

## 3. ESTIMATION AND INFERENCE PROCEDURES

In addition to the challenge of high-dimensional parameters, compounding difficulties are encountered in the low information context where the outcome is sparse, resulting in providers with few readmissions or even no readmission. For very small providers with few patients, there is very low information to assess performance. In extreme cases of providers with no or very low readmission, the FE estimation method [15] leads to unstable estimates for those providers. Thus, in the low information context, we propose a correction to the FE estimates for provider-specific effects.

### 3.1. FE Model Estimation

To describe our proposed FE corrected estimation for provider-specific effects, we first set the notation for the likelihood of the FE model (1) and briefly summarize the alternating Newton-Raphson algorithm proposed by He *et al*. [15]. For the FE model (1), $\text{Pr}(Y_{ij}=1\;|\;Z_{ij})=p_{ij}^{y_{ij}}(1-p_{ij}{)}^{1-y_{ij}}$, and the likelihood function is given by

(3) $$L(\gamma ,\beta )=\prod_{i=1}^{F}\prod_{j=1}^{N_{i}}\frac{\text{exp}\{(\gamma_{i}+\beta^{T}Z_{ij})y_{ij}\}}{1+\text{exp}(\gamma_{i}+\beta^{T}Z_{ij})}.$$

Because direct maximization of (3) is not feasible with standard methods when *F* is large (e.g., *F* ~ 6,000), He *et al*. (2013) proposed an effective iterative algorithm that alternates between estimation of *γ_i_* given *β* and estimation of *β* given *γ_i_* using one-step Newton-Raphson updates. More precisely, estimation of the high-dimensional parameters (*γ*,*β*) are feasible since the likelihood (3) can be written as *L*(*γ*, *β*) = ∏*_i_L_i_*(*γ_i_*, *β*) where *L_i_*(*γ_i_*, *β*) = ∏*_j_* exp{(*γ_i_*, + *β^T^Z_ij_*)*y_ij_*}/x{1 + exp(*γ_i_* + *β^T^Z_ij_*)} for provider *i*. Thus, given *β* , *γ_i_* can be estimated via a Newton-Raphson procedure that depends only on one variable in the maximization of *L_i_*(*γ_i_*,*β*). Briefly, the estimation procedure proposed by He *et al*. (2013) is as follows.

- Set the initial values *β*^(0)^ and $\gamma_{i}^{\left(0\right)}$ of *β* and *γ_i_*, respectively.
- The (*m* + 1) th maximization step for *β* , given $\gamma_{i}^{\left(m\right)}$, is
$$\beta^{\left(m+1\right)}=\beta^{\left(m\right)}+I_{\beta }^{(m{)}^{-1}}U_{\beta }^{\left(m\right)}\;,$$
  where $I_{\beta }^{\left(m\right)}=-\frac{\partial^{2}}{\partial \beta \partial \beta^{T}}\;\log\;L(\gamma^{\left(m\right)},\beta )\;{|}_{{}_{\beta =\beta^{\left(m\right)}}}$ and $U_{\beta }^{\left(m\right)}=\frac{\partial }{\partial \beta }\;\log\;L(\gamma^{\left(m\right)},\beta ){|}_{{}_{\beta =\beta^{\left(m\right)}}}$.
- The (*m* + 1) th maximization step for *γ_i_*, given *β*^(*m*)^, is
$$\gamma_{i}^{\left(m+1\right)}=\gamma_{i}^{\left(m\right)}+I_{i}^{(m{)}^{-1}}U_{i}^{\left(m\right)}$$
  where $I_{i}^{\left(m\right)}=-\frac{\partial^{2}}{\partial \gamma_{i}^{2}}\;\log\;L(\gamma_{i},\beta^{\left(m+1\right)})\;{|}_{{}_{\gamma_{i}=\gamma_{i}^{\left(m\right)}}}$ and $U_{i}^{\left(m\right)}=\frac{\partial }{\partial \gamma_{i}}\;\log\;L(\gamma_{i},\beta^{\left(m+1\right)}){|}_{\gamma_{i}=\gamma_{i}^{\left(m\right)}}$
- The above steps are repeated until convergence, defined by $\max_{i,j}\;|\;p_{ij}^{\left(m+1\right)}-p_{ij}^{\left(m\right)}\;|<\varepsilon$, where $p_{ij}^{\left(m\right)}=g^{-1}(\gamma_{i}^{\left(m\right)}+\beta^{(m{)}^{T}}Z_{ij})$ and *ε* is some prespecified tolerance level. Denote these final uncorrected provider-specific estimates as ${\hat{\gamma }}^{U}=({\hat{\gamma }}_{i}^{U},\ldots ,{\hat{\gamma }}_{F}^{U})$.

Expressions for $I_{\beta }^{\left(m\right)}$, $U_{\beta }^{\left(m\right)}$, $I_{i}^{\left(m\right)}$, and $U_{i}^{\left(m\right)}$ are given in He *et al*. (2013) and they are provided here for convenience: $I_{\beta }^{\left(m\right)}=\sum_{i=1}^{F}\sum_{j=1}^{N_{i}}p_{ij}^{\left(m\right)}(1-p_{ij}^{\left(m\right)})Z_{ij}Z_{ij}^{T}$, $U_{\beta }^{\left(m\right)}=\sum_{i=1}^{F}\sum_{j=1}^{N_{i}}(y_{ij}-p_{ij}^{\left(m\right)})Z_{ij}$, $I_{i}^{\left(m\right)}=\sum_{j=1}^{N_{i}}p_{ij}^{\left(m\right)}(1-p_{ij}^{\left(m\right)})$, and $U_{i}^{\left(m\right)}=\sum_{j=1}^{N_{i}}(y_{ij}-p_{ij}^{\left(m\right)})$. Programs in R, sample data, and tutorial are provided in the online Supplementary Materials. In our implementation, we choose *β*^(0)^ = 0 and $\gamma_{i}^{\left(0\right)}=\log\{{\hat{p}}_{i}^{*}\;∕\;(1-{\hat{p}}_{i}^{*})\}$ where ${\hat{p}}_{i}^{*}=(N_{i}+1{)}^{-1}\{0.5+\sum_{j=1}^{N_{i}}y_{ij}\}$, the Jeffreys’ prior estimated proportion for facility ^i^ (i.e., posterior mean of a Beta distribution, $Beta(0.5+\sum_{j=1}^{N_{i}}y_{ij},0.5.+N_{i}-\sum_{j=1}^{N_{i}}y_{ij}))$.

### 3.2. Corrected Estimation of Provider Effects

As described earlier, estimation of provider effects, *γ_i_* for the FE model can be unstable for some providers in the low information context. Thus, we consider an approach to “correct” or stabilize FE estimates. We adapt the Firth correction in (standard) logistic regression [16, 17] to the high-dimensional FE model (1). Recall that for the standard (non-hierarchical data) logistic regression model with *N* independent units, *j* = 1,…,*N* , Pr(*Y_j_* = 1∣,*Z_j_*,*θ*) = $\{1+\text{exp}(-\sum_{r=1}^{p}Z_{jr}\theta_{r}){\}}^{-1}\equiv \pi_{j}$, where *θ* = (*θ*_1_,…,*θ_p_*) are regression coefficients for covariates *Z_j_* = (*Z*_*j*1_,…,*Z_jp_*). Firth’s modified score equations [16] for estimation to reduce small sample bias is U*(*θ_r_*)≡*U*(*θ_r_*) + 0.5*trace*[*I*(*θ*)^−1^{*∂I*(*θ*)/*∂θ_r_*}] = 0 , for *r* = 1,…, *p*, where *U*(*θ_r_*) = *∂*log *L*/*∂θ_r_*, *I*(*θ*) is the information matrix, and *L* = *L*(*θ*) denotes the likelihood. This is equivalent to using a penalized likelihood L*(θ) = *L*(θ)∣ *I*(θ)∣^−1/2^ [17], where the penalty term ∣*I*(*θ*)^−1/2^ is equivalent to Jeffreys’ prior [18]. Applying this to logistic regression yields the modified estimation equations $U(\theta_{{}_{r}}{)}^{*}=\sum_{j=1}^{N}\{y_{{}_{j}}-\pi_{{}_{j}}+h_{{}_{j}}(0.5-\pi_{{}_{j}})\}Z_{{}_{jr}}=0$ for *r* = 1,…,*p*, with *h_j_* as the *j* th diagonal element of the “hat” matrix *H* = *W*^1/2^*Z*(*Z^T^WZ*)^−1^ *Z^T^W*^1/2^ , with *W* =*diag*{*π*_1_(1 − *π*_1_),…,*π_N_*(1 − *π_N_*)} and *Z* denotes the *N* × *p* data matrix. For binary outcome with small sample size, Firth’s logistic regression has become a standard approach to reduce bias in the estimated regression coefficients.

We adapt this penalized estimation to the high-dimensional FE model (1) to correct for unstable estimation of *γ_i_* for providers with low information. We first note that *β* can be precisely estimated because it is based on data from all providers; therefore, penalization on patient-level risk factors is unnecessary. Direct application of the Firth’s modified score to penalize *γ* = (*γ*_1_,…,*γ_F_*) is not feasible for FE profiling model (1) due to the challenge of calculating the score penalties. These are obtained via the diagonals of the *N* × *N* hat matrix, which in dialysis population applications are in the order of *N* ~ 500,000 or larger. The size of *N* is many orders of magnitude larger for profiling applications in the general population. However, estimating *β* with Firth’s correction, for a fixed *β*, is equivalent to sequentially estimating *γ_i_* individually, for a fixed *β*, using Firth’s correction. This is seen as follows. For a fixed *β*, the hat matrix used in the estimation of *β* with Firth’s correction is *H* = *W*^1/2^*X*(*X^T^WX*)^−1^*X^T^W*^1/2^, where *W* = *W*_1_ ⊕…⊕*W_F_*, *X* = *X*_1_ ⊕…⊕*X_F_*, *W_i_* =*diag*{*p*i*1* (1 − *p*_*i*1_),…, *p_iN_i__*(1 − *p_iN_i__*)} are provider-specific weight matrices, *X_i_* are *N_i_* × 1 provider-specific design matrices of ones, and ⊕ denotes the matrix direct sum operator, e.g., *A* ⊕ *B* is the block diagonal matrix [*A*, 0;0, *B*]. As shown in the Supplementary Appendix section, $H=W^{1∕2}X(X^{T}WX{)}^{-1}X^{T}W^{1∕2}=W_{1}^{1∕2}X_{1}(X_{1}^{T}W_{1}X_{1}{)}^{-1}X_{1}^{T}W_{1}^{1∕2}⊕\cdots ⊕W_{F}^{1∕2}X_{F}(X_{F}^{T}W_{F}X_{F}{)}^{-1}X_{F}^{T}W_{F}^{1∕2}$. Thus, the diagonal of *H* may be obtained sequentially via the diagonals of $W_{i}^{1∕2}X_{i}(X_{i}^{T}W_{i}X_{i}{)}^{-1}X_{i}^{T}W_{i}^{1∕2}$ for each provider *i*.

The *i* th provider hat matrix reduces to $H_{i}=W_{i}^{1∕2}X_{i}(X_{i}^{T}W_{i}X_{i}{)}^{-1}X_{i}^{T}W_{i}^{1∕2}=W_{i}^{1∕2}X_{i}\{(W_{i}^{1∕2}X_{i}{)}^{T}$ $\left(W_{i}^{1∕2}X_{i}){\}}^{-1}(W_{i}^{1∕2}X_{i}{)}^{T}=(w_{i1}^{1∕2},\ldots ,w_{iN_{i}}^{1∕2}{)}^{T}\{(w_{i1}^{1∕2},\ldots ,w_{iN_{i}}^{1∕2}\right)$ $\left(w_{i1}^{1∕2},\ldots ,w_{iN_{i}}^{1∕2}{)}^{T}{\}}^{-1}(w_{i1}^{1∕2},\ldots ,w_{iN_{i}}^{1∕2})\;\;\text{where}\;\;w_{{}_{ij}}=p_{{}_{ij}}(1-p_{{}_{ij}}\right)$. Thus, $diag(H_{i})=(\sum_{j=1}^{N_{i}}w_{ij}{)}^{-1}diag\{(w_{i1}^{1∕2},\ldots ,w_{iN_{i}}^{1∕2}{)}^{T}$ $\left(w_{i1}^{1∕2},\ldots ,w_{iN_{i}}^{1∕2})\}=(w_{i1}\;∕\;\sum_{j=1}^{N_{i}}w_{ij}\;\;,\;\ldots \;,\;\;w_{iN_{i}}\;∕\;\sum_{j=1}^{N_{i}}w_{ij}\right)$ and for a fixed *β*, the estimation of *β* using Firth’s correction can be reduced to a sequence of estimations of a single parameter *γ_i_* by penalizing the score *U_i_*, using the weights $h_{{}_{ij}}=w_{{}_{ij}}\;∕\;\sum_{j=1}^{N_{i}}w_{{}_{ij}}$. More specifically, the provider-specific penalized score equations are $U_{i}^{*}=\sum_{j=1}^{N_{i}}\{y_{{}_{ij}}-p_{{}_{ij}}+h_{{}_{ij}}(0.5-p_{{}_{ij}})\}=0$, for *i* = 1,…,*F*.

We propose a simple correction to adjust the estimates from Section 3.1 of provider-specific effects, *γ_i_*’s, using the modified score $U_{I}^{*}$. More precisely, first, *β* is fixed at the estimate resulting from Section 3.1, namely ${\hat{\beta }}_{U}$. The provider effects *γ_i_*’s are then reestimated using the estimation procedure outlined in 3.1 with the following modifications. In Step (i), *β*^(0)^ is fixed at ${\hat{\beta }}_{U}$ and $\gamma_{i}^{\left(0\right)}$ is set to $\log\;\{{\hat{p}}_{i}\;∕\;(1-{\hat{p}}_{i})\}-N_{i}^{-1}\sum_{j=1}^{N_{i}}{\hat{\beta }}_{U}^{T}Z_{ij}$. Note that when *β*^(0)^ is set to the zero vector, the initial value of *γ*^(0)^ reduces to value previously noted in Step (i) in Section 3.1. In Step (ii), *β*^(*m*+1)^ is set equal to *β*^(*m*)^. In other words, *β* is no longer estimated. Finally, the score in Step (iii) is modified by replacing *U_i_* with $U_{i}^{*}$.

### 3.3. Inference: Identifying Extreme Providers

In profiling, one of the main interests is to identify/flag providers that significantly deviate from the national norm (e.g., national average). The current public policy in the U.S. penalizes providers that perform significantly W than the national standard (SRR >1). Thus, in practice, the goal is to flag/identify providers as W or ND from the national standard (SRR not different than 1). Better (B) providers (SRR <1) are not penalized nor incentivized.

First, note that for a provider with an adjusted event rate that does not differ from the national norm, *γ_i_* = *γ_M_* , which implies *SRR_i_* =1. When *SRR_i_* > 1 or *SRR_i_* < 1, the event rate for provider *i* is greater than or less than the national norm, respectively. Thus, testing the null hypothesis *H*_0_ : *γ_i_* = *γ_M_* is of interest and a test statistic is $T_{i}=\sum_{j=1}^{N_{i}}{\hat{p}}_{ij}$ where ${\hat{p}}_{ij}$ is an estimate of *p_ij_*.

Simultaneously testing the null hypothesis for thousands of providers is computationally expensive. However, one can take advantage of the fact that *β* and *γ_M_* can be estimated based on the large data from all providers. Hence, these parameters are estimated and fixed throughout the proposed algorithm below which is based on resampling responses under the null hypothesis. Since the global parameters *β* and *γ_M_* are fixed, model fitting to the resampled data only requires estimation of provider-level effects *γ_i_* . This reduces the computational burden substantially since each *γ_i_* is estimated using only data from each provider separately. The steps of the procedure for each provider *i* are as follows.

1. Draw *B* samples $\{Y_{ij}^{b}\;:j=1,\ldots ,N_{i}{\}}_{b=1}^{B}$, where each sample and observation is drawn independently from a Bernoulli distribution under the null: $Y_{ij}^{b}∼Bern(g^{-1}\{{\hat{\gamma }}_{{}_{M}}+Z_{ij}^{T}\hat{\beta }\})$, for *b* = 1,…,*B*. (We used *B* = 500 .)
2. Calculate the test statistics for datasets generated/simulated under the null: $T_{i}^{b}=\sum_{j=1}^{N_{i}}{\hat{p}}_{ij}^{b}$ where, ${\hat{p}}_{ij}^{b}=g^{-1}({\hat{\gamma }}_{i}^{b}+Z_{ij}^{T}\hat{\beta })$ and estimation of ${\hat{\gamma }}_{i}^{b}$ only involves steps (iii)-(iv) in Section 3.1 for the uncorrected FE model since *β* is fixed. For the correction method, the estimation proceeds as described earlier in Section 3.2; that is, the corrected estimation algorithm is applied to the b th dataset to obtain ${\hat{p}}_{ij}^{b}$.
3. A nominal two-sided *p*-value for the *i*th provider, *P_i_*, is calculated as
$$P_{{}_{i}}=2\cdot min\left[\begin{array}{c} B^{-1}\sum_{b=1}^{B}\{0.5I(T_{i}^{b}=T_{i}^{O})+I(T_{i}^{b}>T_{i}^{O})\},\\ B^{-1}\sum_{b=1}^{B}\{0.5I(T_{i}^{b}=T_{i}^{O})+I(T_{i}^{b}<T_{i}^{O})\} \end{array}\right],$$
  where $T_{i}^{O}$ is calculated based on the original/observed data and I(A) denotes the indicator function for event A.

## 4. SIMULATION STUDY DESIGN

We designed simulation studies to assess the performance of the uncorrected and corrected FE model estimation methods, mainly with respect to (A) estimation of provider-specific effects, *γ_i_*’s and *SRR_i_*’s; and (B) identification of extreme providers relative to a reference. Data were generated from the model

(4) $$g(\mu_{ij})=\gamma_{{}_{0}}+\gamma_{{}_{i}}+\beta_{{}_{1}}Z_{{}_{ij1}}+\cdots \beta_{{}_{15}}Z_{{}_{ij15}}$$

with *i* = 1,…,*F* = 5,000 providers and *β* = (.25, .25, −.25, −.25, .5, .25, .25, .25, .25, −.25, −.25, −.25,.5,.5,.5)*^T^*. For the patient case-mix vector, *Z_ij_*, the dependence/correlation structure among variables were based on the observed correlations among patient-level variables in real USRDS data. More specifically, Z* is generated from a multivariate normal distribution with means zero and covariance *Cov*(Z*) = *V*^1/2^*RV*^1/2^, where $V^{1∕2}=diag\{\sqrt{Var(Z_{1}^{*})},\ldots ,\sqrt{Var(Z_{15}^{*})}\}$ and *R* is the correlation matrix. The first 5 covariates were taken to be continuous: $Z_{1}\equiv Z_{1}^{*},\ldots ,Z_{5}\equiv Z_{5}^{*}$. The remaining 10 covariates, *Z*_6_,…,*Z*_15_ are binary variables, generated by thresholding corresponding $Z_{{}_{r}}^{{}^{*}}$ so that Pr(*Z*_r_ =1)=*E*(*Z_r_*)’s are equally spaced between 0.2 and 0.8 (for *r* = 6,…,15). The correlation matrix and standard deviations of the 15 variables are provided in the Supplementary Appendix.

For the provider effects, $\{\gamma_{i}{\}}_{i=1}^{F}$, 2.5% were under-performers (W: “worse”) and 2.5% were over-performers (B: “better”) whose effects, *γ_i_*’s, were equally spaced in the intervals [0.4,1.0] and [−1.0, −0.4], respectively. The remaining 95% of providers, with effects not different (ND) from the reference, were generated from a *N*(0,*σ*^2^) distribution with *σ*^2^ = 0.2^2^. Note that a constant *γ*_0_ has been added to simulation model (4) to conveniently control the baseline rate of readmission (outcome data sparsity), where baseline rates of readmission considered were 20%, 10%, 5%, and 3% corresponding to *γ*_0_ = log(1/13.5), log(1/33), log(1/73), and log(1/126), respectively. This setup conveniently regulates the level of outcome data sparsity. For each baseline readmission rate setting, 200 datasets were generated and the estimation (Section 3) and inference procedure (Section 3.3) was applied to each simulated dataset.

The provider volume of each generated dataset range from a minimum of 48 to a maximum of 195 patients on average, similar to real USRDS data in applications (e.g., see [14]). More specifically, the number of patients were generated from a truncated Poisson distribution following He *et al*. (2013), where the number of patients was taken to be $N_{i}=\sum_{{}_{h=1}}^{{}^{1000}}m_{{}_{ih}}1\{m_{{}_{ih}}\leq 7\}$ with $m_{{}_{ih}}∼Poisson(15)$. This process mimics the sparse data structure of dialysis facility (provider) *i* in practice.

## 5. RESULTS

### 5.1. Estimation of Provider-Specific Effects and SRRs

**Figure 1:**
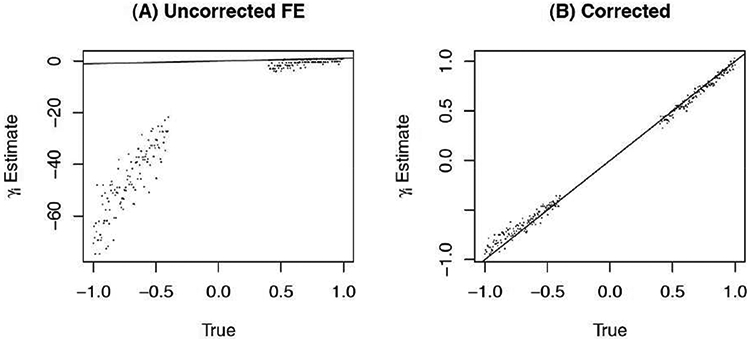
Estimates of provider-specific effects, *γ_i_* < 0 (over-performers) and *γ_i_* > 0 (under-performers) (A) for the uncorrected high-dimensional fixed effects (FE) model and (B) for the corrected method at high-level of outcome data sparsity of 3%. Displayed is average for each *γ_i_* estimate, averaged over 200 simulated data sets.

The results for provider-specific estimates of *γ_i_*’s for the 125 (2.5%) under-performers (*γ_i_* > 0) and 125 over-performers (*γ_i_* <0) for the case of 3% overall event rate (most sparse) are provided in Figure 1A where averages of *γ_i_* estimates over 200 simulated data sets are plotted. As expected, under this extremely low information context, provider effect estimates are unstable for the uncorrected FE method. However, note that these providers are mainly the over-performers (*γ_i_* < 0) with low or zero events ($\sum_{j}y_{ij}’s$ are small) leading to “explosion” of the estimates (Figure 1A). It is important to note that these unstable estimates are in the direction of the true effect (negative direction for negative *γ_i_*’s, where ${\hat{\gamma }}_{i}\to -\infty$). Also as expected, estimates for under-performers (*γ_i_* > 0) are less unstable and more on target for the uncorrected FE method. The corrected estimation approach, which adapts the Firth’s modified score equation for the FE model, largely eliminates the instability and estimates are on more target for the true *γ_i_*’s (Figure 1B).

**Figure 2:**
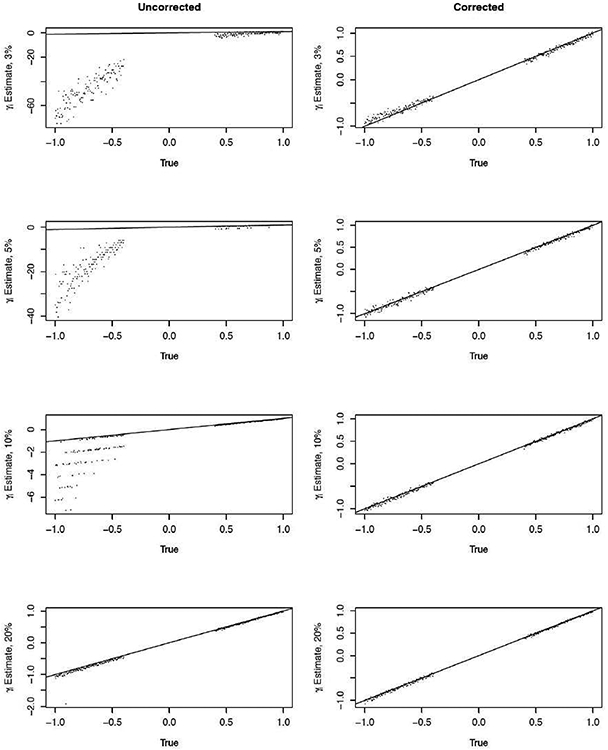
Uncorrected (left column) and corrected (right column) estimation of provider-specific effects, *γ_i_*’s, for 3%, 5%, 10%, and 20% overall outcome event rate. Displayed is average for each *γ_i_* estimate, averaged over 200 simulated data sets.

Figure 2 (left column) shows estimates of *γ_i_*’s for increasing percentage of overall events, from 3% to 20% for the uncorrected FE method. Clearly, the frequency of unstable estimates for *γ_i_* < 0 decreased with increasing overall events, although unstable estimates are apparent even at a 10% event rate. However, the magnitude of the unstable estimates declined quickly (${\hat{\gamma }}_{i}<0$) as the overall event rate increased (e.g., at 20%).

**Figure 3:**
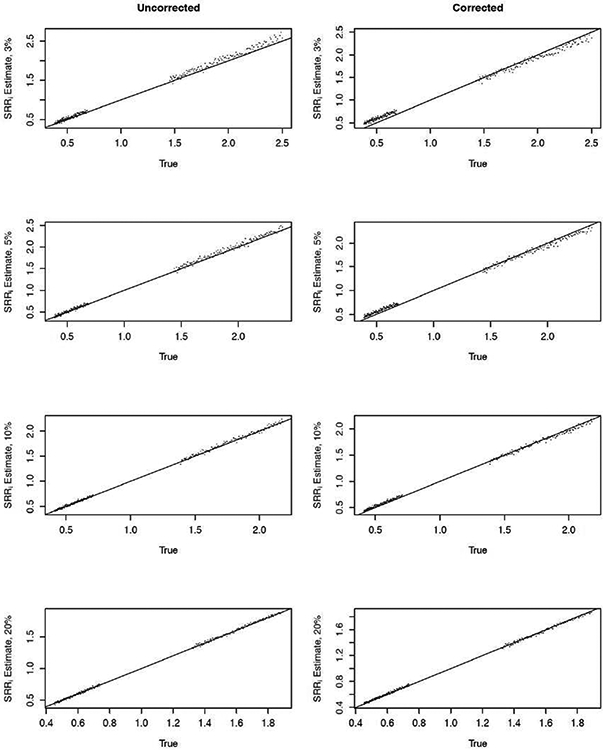
Uncorrected (left column) and corrected (right column) estimates of standardized readmission ratios (SRRs) for 3%, 5%, 10%, and 20% overall outcome event rate. Displayed is average for each *SRR_i_* estimate, averaged over 200 simulated data sets.

**Figure 4:**
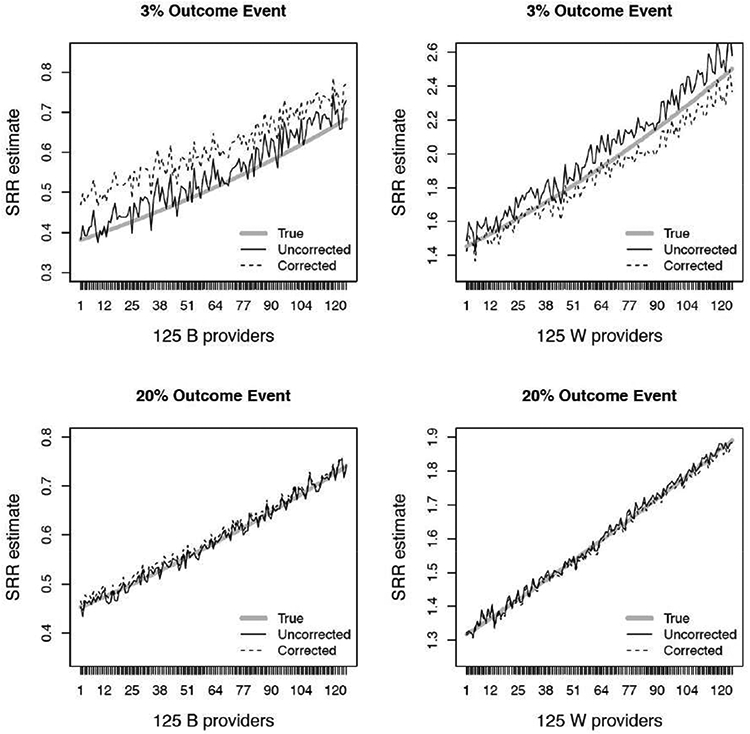
Estimation of standardized readmission ratios (SRRs) for 3% and 20% overall outcome event rate for corrected and uncorrected methods among the 125 better (B) and 125 worse (W) providers. Displayed is average for each *SRR_i_* estimate, averaged over 200 simulated data sets.

Next, we summarize results for estimation of the provider-specific SRRs. As describe in Section 2, SRR is the summary performance index for each provider used in practice which incorporates patient-level risk factors *Z_ij_* and their estimated effects, $\hat{\beta }$. More specifically, given the provider and the patient case-mix effect estimates for each approach, denoted by ${\hat{\gamma }}_{{}_{i}}^{{}^{*}}$ and $\hat{\beta }$, respectively, the estimated SRR for provider *i* is

(5) $$SRR_{i}^{*}=\frac{\sum_{j=1}^{N_{i}}{\hat{p}}_{ij}^{*}}{\sum_{j=1}^{N_{i}}{\hat{p}}_{M,ij}^{*}},$$

where ${\hat{p}}_{{}_{ij}}^{{}^{*}}=g^{-1}({\hat{\gamma }}_{{}_{i}}^{{}^{*}}+{\hat{\beta }}^{T}Z_{{}_{ij}})$, ${\hat{p}}_{{}_{M,ij}}^{{}^{*}}=g^{-1}({\hat{\gamma }}_{{}_{M}}^{{}^{*}}+{\hat{\beta }}^{T}Z_{{}_{ij}})$, * and denotes the uncorrected and corrected approach, namely *U* and *C*. Figure 3 (left column) summarizes the uncorrected FE model estimates of SRR for 3% to 20% overall outcome event. We note that even though specific *γ_i_* < 0 were unstable for highly sparse data (e.g., at 3% - 10%; Figure 2), corresponding estimates of SRR’s are stable overall and targets the true SRR, because SRR incorporates patients characteristics, their effects, as well as provider-specific effects as shown in (5); see Figure 3 (left column). Average SRR estimates for the corrected estimation performed well and are summarized in Figure 3 (right column). However, we note that for extremely sparse data (e.g., at 3%), the uncorrected approach slightly overestimate SRRs while the corrected approach slightly underestimate SRR for truly worse providers (true SRR >1; Figure 4 - top). For truly better providers (true SRR < 1), both methods slightly over estimate the true SRRs, although more so with the corrected method. Differences in SRR estimates between the two methods are neglible as the overall percent of events increases (e.g., at 20%; Figure 4 - bottom).

### 5.2. Flagging Extreme Providers/Facilities

**Figure 5:**
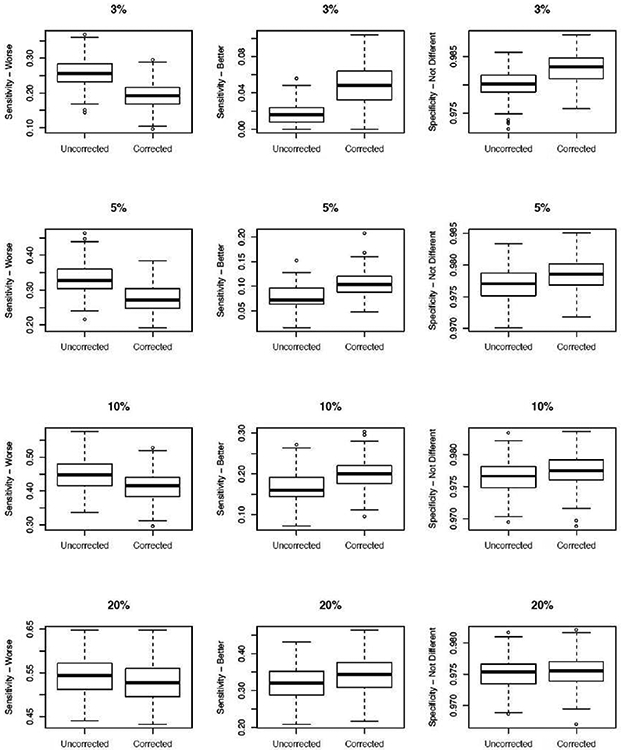
Overall performance of the uncorrected and corrected estimation methods to identify truly worse (sensitivity - worse), truly better (sensitivity - better), and specificity (providers not different from the reference) across data sparsity of 3%, 5%, 10%, and 20% overall outcome event rate. Displayed is average for each *SRR_i_* estimate, averaged over 200 simulated data sets.

The overall performance of the uncorrected and corrected FE methods to identify extreme providers are assessed in terms of sensitivity (SEN) to correctly identify providers that under-perform (W: “worse”), over-perform (B: “better”) relative to the reference standard (e.g., national reference), and specificity. Specificity (SPEC) refers to the correct identification/flagging of providers whose performances are not different from the reference standard (ND: “not different”). We note that provider assessment policies in practice focus on identifying under-performing providers (W providers) as those are tied to payment policy or regulatory goals. Figure 5 summarizes the distribution of SEN-W, SEN-B, and SPEC for varying levels of outcome sparsity, ranging from 3% to 20% overall outcome rate. For extremely sparse data of 3% and 5%, the uncorrected method has highest sensitivity to detect under-performing providers (higher SEN-W; left column). This is expected since the for truly worse providers, there are more outcome events ($\sum_{{}_{j}}y_{{}_{ij}}$); see Figure 5 (left column). SEN-W rates were similar between uncorrected and corrected methods at 20% overall overall outcome rate.

Because the event counts are zero or low for truly better providers in the context of sparse outcome data, the unstable/poor estimation of provider effects from the uncorrected method results in lower sensitivity to detect over-performing providers (lower SEN-B) compared to the corrected method (Figure 5 - middle column). However, note that the nominal SEN-B rates are low overall, as expected, compared to nominal SEN-W rates. This is expected in the low information context since B providers would have fewer readmissions, making it difficult to correctly identify B providers when the outcome is sparse. SPEC rates were high and similar between uncorrected and corrected methods (Figure 5 - right column).

**Figure 6:**
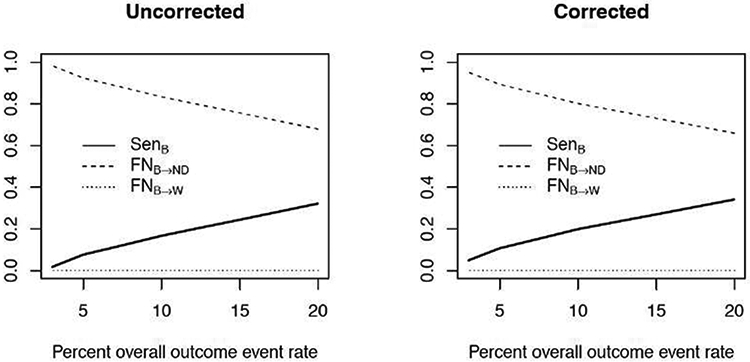
Rate of false negative (FN) for incorrectly flagging better (B) providers as worse (W) providers (*FN_B→W_*) and incorrectly flagging B providers as providers not different (ND) from the reference (*FN_B→ND_*) for the uncorrected and corrected estimation methods across data sparsity of 3%, 5%, 10%, and 20% overall outcome event rate. Displayed is average rates, averaged over 200 simulated data sets.

As mentioned earlier, the main current objective of flagging “extreme” providers in profiling analysis focuses on *identifying W providers and ND providers*. Providers that over-perform (B providers) are not relevant to current payment policy or regulatory objectives. Therefore, under this regime, it is of interest to ensure that there are no (or low rate of) false negatives that misclassify/flag B provider as W provider (*FN_B→W_*). Indeed, there are none, i.e., *FN_B→W_* = 0 across all levels of data sparsity (Figure 6), which is not surprising since W and B providers are on the opposite tails of the distribution of providers. This is true with the uncorrected FE model (as well as the corrected estimation method) since the direction of unstable estimates of *γ_i_*’s are in the same (negative) direction of true *γ_i_* (as pointed out earlier), despite the unstable provider-specific estimates. However, it is not uncommon for false negative classification of a B provider as a ND provider (*FN_B→ND_*). Although *FN_B→ND_* deceases with increasing percentage of overall outcome events as expected, *FN_B→ND_* is common for the extremely low information context (e.g., 3%, 5% overall event rate; Figure 6). We emphasize that high *FN_B→ND_* does not affect current public policy because over-performers are not incentivized and are consider “ND” providers anyway. Therefore, the FE profiling model, even uncorrected, is still useful in the low information context with respect to the current public policy goal of identifying W and ND providers. However, if the public policy goal evolves to also incentivize for better performance, then novel methods able to correctly identify B providers with high sensitivity are needed.

## 6. DISCUSSION

Seminal works by Kalbfleisch and Wolfe [8] and He *et al*. [15] show that FE model estimates have smaller error for outlier providers whose effects are exceptionally large or small, and these extreme providers are precisely the ones we wish to identify in profiling analysis. The high-dimensional FE models were then used to assess the performance of dialysis facilities (providers) with respect to all-cause hospital readmissions which are frequent outcomes in dialysis patients. Subsequently, our own works have elucidated several operating characteristics [9, 10] of the FE profiling models and have been applied to assess the performance of dialysis facilities with respect to all-cause 30-day readmissions [11, 12, 14]. However, to date there is no work that examines the performance of FE models in the low information context where the outcome is sparse. The current study starts to fill this gap in knowledge. Several findings from this study have important practical impact in the low information context. First, even though the provider-specific estimates with true *γ_i_* < 0 (truly B providers) are unstable, they are in the same direction as the true effects and the instability has moderated effects on the estimation of SRRs; i.e., SRRs are reasonably well-estimated and are the relevant quantities used in practice as they incorporate patient case-mix. However, if the provider-specific estimates, ${\hat{\gamma }}_{{}_{i}}’s$, are themselves of interest, then our proposed correction method can be used to provide better estimates, especially corresponding to uncorrected ${\hat{\gamma }}_{{}_{i}}$ that are substantially less than zero. Second, the consequence of sparse outcome data impacts more directly inference for B providers because true over-performers are the ones that contribute no or few events (readmissions); however, this “deficit” in estimation does not greatly impact the identification of W providers/under-performers and ND providers, which is the current focus of profiling in practice. Development of novel methods that have better sensitivity for flagging B providers would be useful when public policies or regulatory goals incorporate an incentive for over-performers.

## Supplementary Material

Supplementary material

## APPENDIX

### Provider-Specific Modified Score Equation Penalties

We describe details of calculating the penalties for the Firth’s modified score equations, adapted for the high-dimensional FE profiling model in Section 3.2. For a fixed *β*, the hat matrix used in the estimation of *γ* with Firth’s correction is *H* = *W*^1/2^*X*(*X^T^WX*)^−1^*X^T^W*^1/2^ where *W* = *W*_1_ ⊕…⊕*W_F_*, *X* = *X*_1_ ⊕…⊕*X_F_*, *W_i_* =*diag*{*p*_*i*1_(1−*p*_*i*1_),…,*p_iN_i__*(1−*p_iN_i__*)} are provider-specific weight matrices (see Section 3.2). Direct calculation yields

$$H=W^{1∕2}X(X^{T}WX{)}^{-1}X^{T}W^{1∕2}\;=W^{1∕2}X\{(X_{1}⊕\cdots ⊕X_{F}{)}^{T}\;(W_{1}⊕\cdots ⊕W_{F})(X_{1}⊕\cdots ⊕X_{F}){\}}^{-1}X^{T}W^{1∕2}\;=W^{1∕2}X(X_{1}^{T}W_{1}X_{1}⊕\cdots ⊕X_{F}^{T}W_{F}X_{F}{)}^{-1}X^{T}W^{1∕2}\;=W^{1∕2}X\{(X_{1}^{T}W_{1}X_{1}{)}^{-1}⊕\cdots ⊕(X_{F}^{T}W_{F}X_{F}{)}^{-1}\}X^{T}W^{1∕2}\;=(W_{1}^{1∕2}X_{1}⊕\cdots ⊕W_{F}^{1∕2}X_{F})\{(X_{1}^{T}W_{1}X_{1}{)}^{-1}⊕\cdots ⊕(X_{F}^{T}W_{F}X_{F}{)}^{-1}\}X^{T}W^{1∕2}\;=\{W_{1}^{1∕2}X_{1}(X_{1}^{T}W_{1}X_{1}{)}^{-1}⊕\cdots ⊕W_{F}^{1∕2}X_{F}(X_{F}^{T}W_{F}X_{F}{)}^{-1}\}X^{T}W^{1∕2}\;=\{W_{1}^{1∕2}X_{1}(X_{1}^{T}W_{1}X_{1}{)}^{-1}⊕\cdots ⊕W_{F}^{1∕2}X_{F}(X_{F}^{T}W_{F}X_{F}{)}^{-1}\}(X_{1}^{T}W^{1∕2}⊕\cdots ⊕X_{F}^{T}W^{1∕2})\;=W_{1}^{1∕2}X_{1}(X_{1}^{T}W_{1}X_{1}{)}^{-1}X_{1}^{T}W_{1}^{1∕2}⊕\cdots ⊕W_{F}^{1∕2}X_{F}(X_{F}^{T}W_{F}X_{F}{)}^{-1}X_{F}^{T}W_{F}^{1∕2}\;.$$

Thus, $diag(H)=[diag\{W_{1}^{1∕2}X_{1}(X_{1}^{T}W_{1}X_{1}{)}^{-1}X_{1}^{T}W_{1}^{1∕2}\},\ldots ,diag\{W_{F}^{1∕2}X_{F}(X_{F}^{T}W_{F}X_{F}{)}^{-1}X_{F}^{T}W_{F}^{1∕2}\}]$.

### Dependence Structure of Covariates in Simulation Model

The correlation matrix and the standard deviation of the patient case-mix variables, *Z*_*ij*1_,…,*Z*_*ij*15_, are summarized in Table 1.

**Table 1:** Correlation Matrix of *Z*_1_,…,*Z*_15_ and their Standard Deviations

Correlation														
*Z*_1_	*Z*_2_	*Z*_3_	*Z*_4_	*Z*_5_	*Z*_6_	*Z*_7_	*Z*_8_	*Z*_9_	*Z*_10_	*Z*_11_	*Z*_12_	*Z*_13_	*Z*_14_	*Z*_15_
	−0.06	−0.01	−0.31	0.04	0.08	−0.12	−0.05	0.28	−0.04	−0.13	−0.22	−0.06	0.02	−0.01
	1	0.03	−0.16	0.20	0.33	−0.05	0.08	0.07	0.01	−0.04	−0.05	−0.04	−0.05	0.00
		1	0.00	0.03	0.03	0.00	0.10	0.00	0.07	0.08	0.01	0.02	0.02	0.10
			1	−0.01	−0.67	0.00	0.09	−0.03	0.11	0.17	0.04	0.01	0.04	0.04
				1	0.06	0.00	−0.10	0.02	0.05	0.02	−0.06	−0.09	0.02	0.01
					1	−0.07	0.21	−0.03	−0.01	−0.12	−0.04	−0.02	−0.06	0.01
						1	−0.02	−0.03	0.00	0.07	0.06	0.25	0.02	−0.02
							1	0.06	0.13	0.09	0.06	0.02	−0.01	0.08
								1	0.48	0.15	0.11	0.06	0.18	0.06
									1	0.33	0.17	0.07	0.19	0.14
										1	0.21	0.29	0.10	0.10
											1	0.34	0.03	0.03
												1	0.02	0.01
													1	0.01
														1
	
Standard Deviation														
1.59	1.03	0.87	1.43	0.50	0.49	0.10	0.24	0.41	0.36	0.32	0.20	0.19	0.16	0.19

## LIST OF ABBREVIATIONS

CMS: Centers for Medicare and Medicaid Services
FE: Fixed effects
US: United States
USRDS: United States Renal Data System
RE: Random effects
SRR: Standardized readmission ratio
W: Worse
ND: Not different
B: Better
SEN: Sensitivity
SPEC: Specificity

## References

[1] United States Renal Data System. Annual Data Report: Epidemiology of Kidney Disease and in the United States. National Institutes of Health, National Institute of Diabetes and Digestive and Kidney Diseases, Bethesda, MD. [cited 2020]: Available from https://adr.usrds.org/2020/

[2] Ash AS, Fienberg SE, Louis TA, Normand ST, Stukel TA, Utts J. Statistical issues in assessing hospital performance. The COPSS-CMS White Paper Committee, CMS, Washington D.C. [cited 2012]: Available from https://www.cms.gov/medicare/quality-initiatives-patient-assessment-instruments/hospitalqualityinits/downloads/statistical-issues-in-assessing-hospital-performance.pdf.

[3] Centers for Medicare & Medicaid Services (CMS)/UM-KECC. Report for the standardized readmission ratio. [cited 2017]: Available from https://www.cms.gov/Medicare/Quality-Initiatives-Patient-Assessment-Instruments/ESRDQIP/Downloads/SRR_Methodology_Report_June2017.pdf

[4] Horwitz L, Partovain C, Lin ZQ, Development and use of an administrative claims measure for profiling hospital-wide performance on 30-day unplanned readmission. Annals of Internal Medicine 2014; 161: S66–75. 10.7326/M13-3000 25402406 PMC4235629

[5] Krumholz HM, Lin Z, Drye EE, An administrative claims measure suitable for profiling hospital performance based on 30-day all-cause readmission rates among patients with acute myocardial infarction. Circulation Cardiovascular Quality and Outcomes 2011; 4: 243–252. 10.1161/CIRCOUTCOMES.110.957498 21406673 PMC3350811

[6] Normand S, Glickman ME, Gatsonis CA. Statistical methods for profiling providers of medical care: Issues and applications. Journal of the American Statistical Association 1997; 92: 803–814. 10.1080/01621459.1997.10474036

[7] Normand ST, Shahian DM. Statistical and clinical aspects of hospital outcomes profiling. Statistical Science 2007; 22: 206–226. 10.1214/088342307000000096

[8] Kalbfleisch JD, Wolfe RA. On monitoring outcomes of medical providers. Statistics in Biosciences 20113; 5: 286–302. 10.1007/s12561-013-9093-x

[9] Chen Y, Senturk D, Estes JP, Campos LF, Rhee CM, Dalrymple LS, Kalantar-Zadeh K, Nguyen DV. Performance characteristics of profiling methods and the impact of inadequate case-mix adjustment. Communications in Statistics - Simulation and Computation 2021; 50: 1854–1871. 10.1080/03610918.2019.1595649 PMC7731974 33311840

[10] Senturk D, Chen Y, Estes JP, Campos LF, Rhee CM, Kalantar-Zadeh K, Nguyen DV. Impact of case-mix measurement error on estimation and inference in profiling of health care providers. Communications in Statistics - Simulation and Computation 2020; 49: 2206–2224. 10.1080/03610918.2018.1515360 33311842 PMC7731965

[11] Estes JP, Chen Y, Senturk D, Rhee CM, Kurum E, You AS, Streja E, Kalantar-Zadeh K, Nguyen DV. Profiling dialysis facilities for adverse recurrent events. Statistics in Medicine 2020; 39: 1374–1389. 10.1002/sim.8482 31997372 PMC7125020

[12] Estes JP, Nguyen DV, Chen Y, Dalrymple LS, Rhee CM, Kalantar-Zadeh K, Senturk D. Time-dynamic profiling with application to hospital readmission among patients on dialysis (with discussion). Biometrics 2018; 74: 1383–1394. 10.1111/biom.12908 29870064 PMC6296887

[13] Estes JP, Nguyen DV, Chen Y, Dalrymple LS, Rhee CM, Kalantar-Zadeh K, Senturk D. Rejoinder: Time-dynamic profiling with application to hospital readmission among patients on dialysis. Biometrics 2018b; 74: 1404–1406. 10.1111/biom.12905 29870066 PMC6296889

[14] Chen Y, Rhee CM, Senturk D, Kurum E, Campos LF, Li Y, Kalantar-Zadeh K, Nguyen DV. Association of U.S. dialysis facility staffing with profiling of hospital-wide 30-day unplanned readmission. Kidney Diseases 2019; 5: 153–162. 10.1159/000496147 31259177 PMC6587206

[15] He K, Kalbfleisch JD, Li Y, Li Y. Evaluating hospital readmission rates in dialysis facilities; adjusting for hospital effects. Lifetime Data Analysis 2013; 19: 490–512. 10.1007/s10985-013-9264-6 23709309

[16] Firth D Bias reduction of maximum likelihood estimates. Biometrika 1993; 80: 27–38. 10.1093/biomet/80.1.27

[17] Heinze G, Schemper M. A solution to the problem of separation in logistic regression. Statistics in Medicine 2002; 21: 2409–2419. 10.1002/sim.1047 12210625

[18] Jeffreys H An invariant form of the prior probability in estimation problems. Proceedings of the Royal Society of London, Series A 1946; 186(1007): 453–461. 10.1098/rspa.1946.0056 20998741

