# Supplementary material for: Fixed Effects High-Dimensional Profiling Models in Low Information Context
Source: Int J Stat Med Res. Author manuscript; Available in PMC 2022 Apr 22. (PMC9033160; doi:10.6000/1929-6029.2021.10.11)
Supplement: Supplementary material [file NIHMS1745804-supplement-Supplementary_material.pdf]

Supplementary Materials

Fixed Effects High-Dimensional Profiling Models in Low Information Context

Jason P. Estes[1], **Damla Şentürk**[2], **Esra Kürüm**[3], **Connie M. Rhee**[4], **and Danh V. Nguyen**[4] *

[1]Mountain View, CA 94043, U.S.A.
[2]Department of Biostatistics, University of California, Los Angeles, CA 90095, U.S.A.
[3]Department of Statistics, University of California, Riverside, CA 92521, U.S.A.
[4]Department of Medicine, University of California Irvine, Orange, CA 92868, U.S.A.

*Address correspondence this author at Department of Medicine, University of California Irvine, 333 City Blvd. West, City Tower, Suite 400 Orange, CA 92868, USA ; E-mail: danhvn1@hs.uci.edu

1 Analysis Example and R Codes

This supplemental materials document provides a tutorial on the implementation of the uncorrected (Section 3.1) and corrected (Section 3.2) model fitting procedures in addition to their corresponding flagging procedures (Section 3.3) based on the nominal p-values and empirical null adjustment. A simulated example dataset following the simulation setup outlined in Section 4 along with R codes are also provided. The example dataset and R codes can be downloaded at https://sites.google.com/view/usrds-modeling/software. The example data set is the R data file `data_example_low_information.Rdata`, the R codes for model fitting procedures and hypothesis testing procedures are located in the file `low_information_procedures.R`, and the analysis script that runs the models are in the file `low_information_analysis.R`.

1.1 Example Data

The example dataset (563,067 rows x 17 columns) contained 5,000 providers and 15 patient case-mix covariates with an overall event rate of 5% (mean of outcome y). The example data consists of 5 continuous and 10 binary case-mix covariates (see Section 4 of the main paper). See the header of dataset (`df`) below.

```
> load("data_example_low_information.Rdata")
> head(df)
  y fid         z1         z2         z3         z4          z5 z6 z7 z8 z9 z10 z11 z12 z13 z14 z15
1 0   1  0.2759839 -0.7518953 -0.4472231  0.3351318 -0.37483191  0  0  0  0   0   1   1   1   1   1
2 0   1 -1.9049438 -0.6313709  0.5496096  1.1532731  0.57002474  0  1  0  1   1   0   1   1   1   0
3 0   1  1.9290079 -1.0494348  0.5849342  0.4836403  0.47714465  1  0  0  0   1   1   1   1   1   1
4 0   1 -0.6758763  0.2443143  0.8313176 -0.7210276  0.73505279  0  0  1  0   0   0   1   1   1   0
5 0   1  5.3987338 -1.1921968  0.8750204 -1.3041801  0.06302962  1  0  1  0   1   0   1   1   0   0
6 0   1 -2.3464001  0.5910203  2.1762631  2.0819778  1.07015106  0  1  0  1   1   0   1   1   1   1
```

Here are the conventions for running the codes:

1. The outcome variable is labelled '`y`' (binary 0, 1=event).

2. The facility (provider ID) is labelled '`fid`'. The model fitting algorithm/code assumes that the dataset is ordered by facility/provider ID (`fid`) and this ID must be integer values between 1 and $F = 5000$, inclusively.

3. The covariates are named ‘z1’, ‘z2’ etc. with categorical variables converted to binary (0, 1=event; e.g., dummy variable/reference coding). The example codes assume 15 covariates (z1, ..., z15) as in the example data structure. To accommodate other number of covariates, modify the first line of the functions `fitModelUncorrected`, `fitModelCorrected`, `getPValuesCorrected`, and `getPValuesCorrected`. Specifically, change

   `cov_names=paste( c('z'), 1:15, sep="" )`

   to accommodate different number of covariates.

1.2 Implementation of Uncorrected and Corrected Fitting Procedures

The following functions implement the uncorrected and corrected fitting procedures respectively.

```
fitModelUncorrected(df, convTol=1e-8, maxIter=500)
fitModelCorrected(df, fit_obj_uncorrected, convTol=1e-8, maxIter=500)
```

`df` is the sample dataset (“`data_example_low_information.Rdata`”), `fit_obj_uncorrected` is the uncorrected fit object returned by the function `fitModelUncorrected`, `convTol` is the value used to determine convergence (default value is 1e-8), and `maxIter` is the maximum number of iterations attempted for convergence (default value is 500).

1.3 Implementation of Flagging Procedures

The following implements the uncorrected and corrected flagging procedures respectively.

```
flaggingUncorrected(df, fit_obj_uncorrected, numBS=500, convTol=1e-8, maxIter=500)
flaggingCorrected(df, fit_obj_corrected, numBS=500, convTol=1e-8, maxIter=500)
```

`numBS` is the number of resamples (default value is 500) for testing the hypothesis $H_0 : \gamma_i = \gamma_M$.

1.4 Output Objects from Procedures

Both the uncorrected and corrected fitting procedures output a list with the following objects

```
> names(fit_uncorrected)
[1] "betaEst"    "gammaEst"    "sRRN"    "sRRD"    "sRREst"    "convergence"    "numIter"
```

containing the 15 regression coefficient estimates (`betaEst`), the 5000 provider effect estimates (`gammaEst`), the numerator of SRR for each of the 5000 providers (`sRRN"`), the denominator of SRR for each of the 5000 providers (`sRRD`), SRR for each of the 5000 providers (`sRREs`), an indicator of whether convergence criteria was met (`convergence`), and the number of iterations the fitting algorithm ran (`numIter`).

The flagging procedures each output a data frame with the following columns names

```
colnames(flagging_uncorrected)
[1] "sRREst"   "gammaEst"   "pVal.nom"   "sRR.Category.emp"
```

1. `sRREst`: SRR_i for $i = 1, 2, ..., 5000$
2. `gammaEst`: $\widehat{\gamma}_i$ estimate for each provider.
3. `pVal.nom`: Nominal P-values from hypothesis test of $H_0 : \gamma_i = \gamma_M$, $i = 1, 2, \ldots, 5000$.
4. `SRR.category.nom`: Flagging labels based on the nominal P-values (ND: not different, B: better, W: worse).
5. `SRR.category.emp`: Flagging labels based on the empirical null distribution (ND: not different, B: better, W: worse).

The output header is given below.

```
> head(flagging_uncorrected)
     sRREst  gammaEst pVal.nom SRR.category.nom SRR.category.emp
1 0.8214732 -4.493083    0.756               ND               ND
2 0.7777746 -4.550121    0.568               ND               ND
3 0.4166257 -5.194318    0.170               ND               ND
4 0.2313673 -5.808389    0.004                B                B
5 0.5268401 -4.957354    0.168               ND               ND
6 1.0805570 -4.190823    0.778               ND               ND
```
